# Characterization of *Boeremia exigua* causing stem necrotic lesions on Luobuma in northwest China

**DOI:** 10.1038/s41598-022-25125-1

**Published:** 2022-12-14

**Authors:** Yanru Lan, Tingyu Duan

**Affiliations:** 1 State Key Laboratory of Herbage Improvement and Grassland Agro-Ecosystems, Lanzhou University, Lanzhou, 730020 China; 2 Key Laboratory of Grassland Livestock Industry Innovation, Ministry of Agriculture and Rural Affairs, Lanzhou, 730020 China; 3 College of Pastoral Agriculture Science and Technology, Lanzhou University, Lanzhou, 730020 China

**Keywords:** Microbiology, Molecular biology

## Abstract

Luobuma (*Apocynum venetum*, *Poacynum pictum*, and *P. hendersonni*) are perennial herbs widely used in the textile and medical industries and ecological restoration. In the summer of 2020, reddish-brown or off-white sunken shape necrotic lesions were observed on the stems and shoots of seven Luobuma ecotypes grown in the field in Yuzhong County, Gansu province of China, which is a limiting factor that affects the growth, function and application of Luobuma. To make clear whether the new symptoms were caused by a novel pathogen, a combined research in field and greenhouse was conducted. Based on the morphological and molecular analysis results, the pathogen causing the necrotic lesions was identified as *Boeremia exigua* var. *rhapontica*. The incidence and disease index of the seven ecotypes in the field ranged from 11.49 to 33.68% and 6.63 to 23.01, respectively, from 2020 to 2021. The results showed that the disease severity gradually increased with the growing season. According to the pathogenicity analysis of the eight ecotypes in the greenhouse, the ecotypes Pp-BMK and Pp-BMH were susceptible, while ecotype Pp-BMQ was resistant to *Boeremia exigua* var. *rhapontica* infection. Thus, the present study provides a theoretical basis for preventing and controlling the stem and leaf necrotic lesions disease on Luobuma by planting resistant varieties/ecotypes. To our knowledge, this is the first report of stem necrotic lesions and leaf spots on Luobuma caused by *B. exigua* var. *rhapontica*.

## Introduction

Luobuma plants are popular traditional herbs belonging to the family Apocynaceae^1^ and are widely distributed in Central Asia (Caspian Sea, Kazakhstan, Turkmenistan, Uzbekistan, and Northwest China)^2^. Similar to the wild Luobuma, which are mostly found in steppes, semi-deserts, and deserts zones^2^, cultivated Luobuma are also adapted to arid climates or dry areas with moderate salinity^2^. Luobuma plants are increasingly becoming important economic crops worldwide due to their wide usage in tea, medicine, and textiles^1^.

Luobuma plants include two genera with three species; *Apocynum venetum*, *Poacynum pictum,* and *P. hendersonii*^3^. In 2017, eight Luobuma ecotypes were introduced to Yuzhong, Gansu province from Xinjiang, China^4^. Taxonomic and phenotypic identification of the eight ecotypes was conducted by Gao et al.^5^. Among these ecotypes, *P. hendersonii* plants got extinct in 2019, leaving only seven ecotypes in Yuzhong, Gansu province, China, to date. A novel necrotic lesions symptom has been frequently observed on the stem of the different ecotypes in Yuzhong in 2020. The fungal isolations from these stems have been identified as *Boeremia exigua* (synonym: *Phoma exigua*)^6^, based on their morphology, spores, and hypha of fungus colony.

*Boeremia exigua* is a plant pathogen causing serious leaf (petioles and rachises), stem, or root lesion diseases on various plants worldwide. Examples of diseases caused by this pathogen include leaf necrotic lesions disease of *Fraxinus excelsior* in Poland^7^, snags rotten leaves disease of *Morus malba* in China^8^, leaf spot of *Hydrangea paniculate* in Italy^9^, black root rot of *Cichorium intybus* var. *sativum* in Chile^10^, and leaves necrotic lesions of *Cycas circinalis* in India^11^. *Boeremia exigua* species has 11 varieties, which are *B. exigua* var. *coffeae*, *B. exigua* var. *exigua*, *B. exigua* var. *heteromorpha*, *B. exigua* var. *pseudolilacis*, *B. exigua* var. *linicola*, *B. exigua* var. *forsythiae*, *B. exigua* var. *viburni*, *B. exigua* var. *gilvescens*, *B. exigua* var. *populi*, *B. exigua* var. *inoxydabilis*, and *B. exigua* var. *rhapontica*^12,13^. These varieties are mainly distinguished based on their host specificity and pathogenicity^12^, and molecular methods are often used to support the morphological identifications^12,13^. Previous studies have found that the genus *Boeremia* could be successfully identified based on its actin, beta-tubulin, calmodulin, elongation factor, and *ITS* genes^7,12,13^.

Stem necrotic lesions disease was observed in Luoboma fields in 2020, with Luobuma ecotypes with severe disease symptoms. Thus, the present study aimed to (i) identify the pathogens causing the necrosis lesion symptoms of Luobuma and (ii) test the pathogenicity and occurrence of the pathogen on different ecotypes in the field.

## Results

### Symptoms of stem necrotic lesions disease on Luobuma in the field

**Figure 1:**
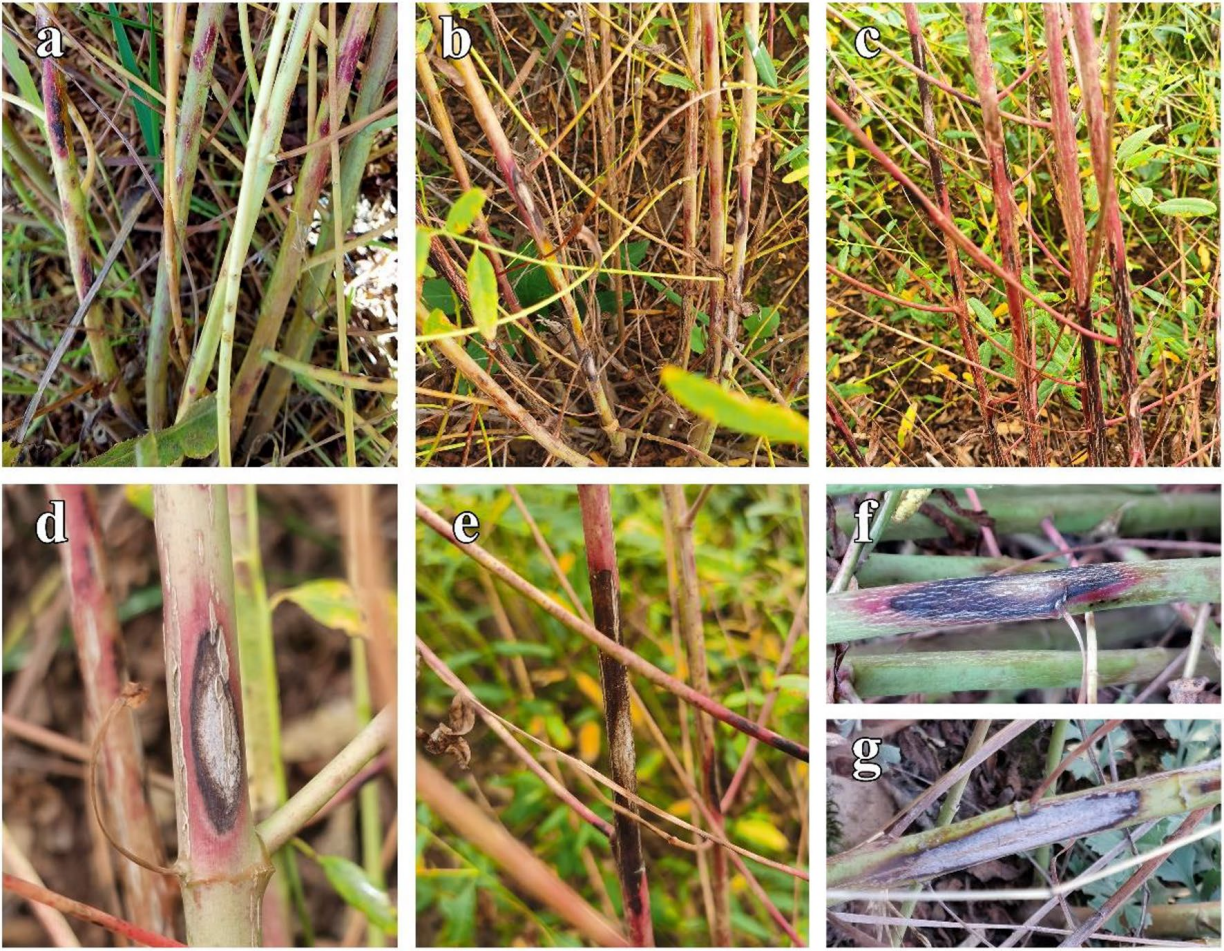
The symptom of stem necrosis lesions disease on Luobuma caused by *B. exigua* in the field. (**a**) early stage of stem necrosis lesion, (**b**) mid stage of stem necrosis lesion, (**c**) late stage of stem necrosis lesion and (**d–f**) the detail symptoms of stem necrosis lesion spot.

Leaf symptoms caused by the pathogen *Boeremia exigua* were not observed on Luobuma plants; however, serious necrotic lesion symptoms were observed on the stem between 2020 and 2021. The initial symptom on stems and shoots began as small reddish-brown spots, with an unclear or clear borderline between infected and healthy tissues. The spot sizes later enlarged, forming irregular, rhomboid, diamond, or sunken shapes, with white or lighter coloured centers. However, when the stem necrotic lesions became severe, the spots turned white or off-white with ripe pycnidia girdling the stems forming depressions, which then withered and died (Fig. 1).

### Isolation frequency of *Boeremia exigua* strain

We isolated 38 strains from the 70 plants of the seven ecotypes with stem necrotic lesion symptoms. The isolation frequency of the strains from the infected stem tissues and pycnidia on the infected stem tissues were 42.5% and 100%, respectively. The pathogen was not found in the root tissues (without symptoms) of the infected plants but was isolated successfully from the soil. Thus, eight strains (seven from the seven ecotypes and one from the soil) were screened for further analysis (Table 1).

**Table 1:** The pathogens codes, isolation sources, and the ecotype codes of corresponding species of eight strains used in this study.

Pathogens codes	Isolation sources	Ecotypes codes	Plant species
AvLM-Be	Stem	Av-LHM	*Apocynum venetum*
PpBQ-Be	Stem	Pp-BMQ	*Poacynum pictum*
PpBB-Be	Stem	Pp-BMB	*P. pictum*
PpBH-Be	Stem	Pp-BMH	*P. pictum*
PpBZ-Be	Stem	Pp-BMZ	*P. pictum*
PpBX-Be	Stem	Pp-BMX	*P. pictum*
PpBK-Be	Stem	Pp-BMK	*P. pictum*
Av-Be	Soil	–	–

For each strain, a spore was transferred onto a fresh PDA plate under a compound microscope (YS2-H, NIkon, China) to obtain purified pathogens for further analysis.

### Morphological and biological characteristics

**Figure 2:**
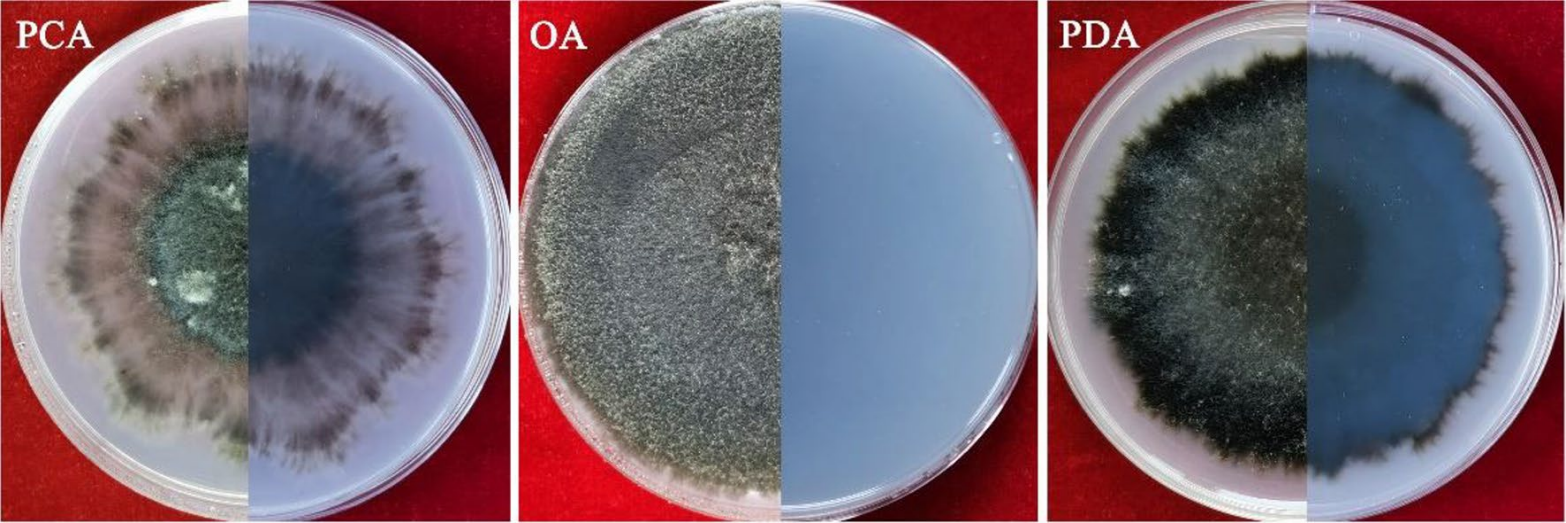
The colony of *B. exigua* cultured in the PCA, OA, and PDA.

**Figure 3:**
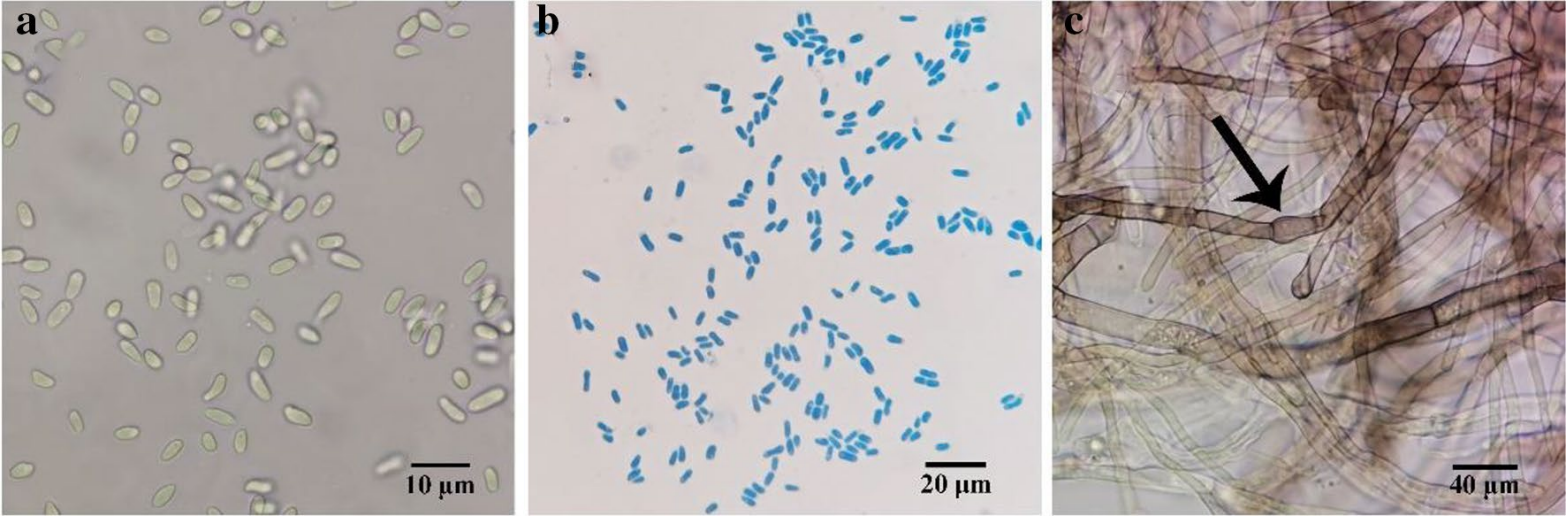
Morphological characteristics of the pathogen cultured in the PCA at 25 °C for 20 days. (**a,b**) conidia and (**c**) mycilium.

The morphological characteristics of the eight pathogenic strains were consistent. The colonies grew to 39 mm in diameter on OA, 44 mm on PDA, and 24 mm on PCA 5 days after inoculation. After 10 days, the colonies had 53 mm diameter on OA, 69 mm on PDA, and 48 mm on PCA; however, those on PCA were irregular, salmon with light brown aerial mycelium, and had scattered growth with visible fascicular branches. Colonies on OA were regular, white/olivaceous to olivaceous with olivaceous aerial mycelium, while those on PDA were regular and tan/light salmon panniform-like colonies with developed white/olivaceous aerial mycelium. However, the colonies had irregular whitish-grey lobed margins on PCA, OA, and PDA (Fig. 2). Conidia on PCA were hyaline, smooth, oblong with obtuse ends, aseptate, straight, sometimes guttulate, and measured 4.94–10.13 μm × 2.03–3.01 μm (average 2.67 μm × 6.42 μm) (Fig. 3a,b). Moreover, the hyphae on PCA were brown, septate, synaxillary branches which were mostly binary at acute angles, with node-like expansion (Fig. 3c). Thus, the pathogen was preliminarily identified as *Boeremia exigua* based on the morphological characteristics.

### Molecular identification and phylogenetic analysis

To verify the accuracy of the morphological identification, we sequenced 8 strains screened in this study and obtained the sequences of 24 strains (6 species and 11 varieties) including *Phoma herbarum* as the outgroup from NCBI for the phylogenetic analysis (Table 2).

**Table 2:** Species/varieties, strain numbers and GenBank accessions were indicated in this study.

Species	Strain number	Hosts	Genbank accessions			
			*ITS*	*ACT*	*TUB*	*TEF*
*Boeremia exigua* var. *heteromorpha*	CBS 101196	*Nerium oleander* (Apocynaceae)	KY484660.1	EU880870.1	GU237496.1	KY484699.1
	CBS 443.94		GU237866.1	EU880869.1	GU237497.1	KY484700.1
*B. exigua* var. *populi*	CBS 100168	*Populus euramerica* (Salicaceae)	KY484659.1	EU880886.1	MN983801.1	KY484707.1
*B. exigua* var. *linicola*	CBS 109.49	*Linum usitatissimum* (Linaceae)	KY484657.1	EU880877.1	KY484755.1	KY484702.1
	CBS 116.76		GU237754.1	EU880880.1	GU237500.1	KY484705.1
	CBS 112.28		MH854942.1	EU880878.1	KY484756.1	KY484703.1
*B. exigua* var. *exigua*	CBS 101201	*Plox* sp. (Polemoniaceae)	KY484641.1	KY484561.1	MN983753.1	KY484682.1
*B. exigua var. forsythiae*	CBS 101213	Forsythia sp. (Oleaceae)	GU237723.1	EU880868.1	GU237494.1	KY484692.1
	CBS 101197		GU237718.1	EU880866.1	MN983731.1	KY484690.1
*B. exigua* var. *viburni*	CBS 101211	*Viburnum opulus* (Capritoliaceae)	GU237722.1	EU880891.1	GU237507.1	KY484711.1
*B. exigua* var. *inoxydabilis*	CBS 372.75	*Vinca minor* (Apocynaceae)	KY484656.1	KY484565.1	KY484754.1	KY484701.1
*B. exigua var. pseudolilacis*	CBS 101207	*Syringa vulgaris* (Oleaceae)	GU237721.1	EU880874.1	GU237503.1	KY484710.1
*B. exigua* var. *coffeae*	CBS 109183	*Coffea arabica* (Rubiaceae)	GU237748.1	KY484560.1	GU237505.1	KY484678.1
*B. exigua* var. *gilvescens*	PD98/213	*Veronica* sp. (Scrophulariaceae)	KY484653.1	KY484563.1	KY484752.1	KY484698.1
*B. diversispora*	CBS 102.80	*Phaseolus vulgaris* (Fabaceae)	GU237725.1	EU880861.1	GU237492.1	KY484676.1
	CBS 101194		GU237716.1	EU880864.1	GU237491.1	KY484674.1
*B. noackiana*	CBS 101215	*Phaseolus vulgaris* (Fabaceae)	KY484671.1	EU880883.1	MN983821.1	KY484730.1
	CBS 101216		KY484672.1	EU880884.1	MN983822.1	KY484731.1
*B. strasseri*	CBS 126.93	*Mentha* sp. (Lamiaceae)	GU237773.1	EU880904.1	GU237518.1	KY484735.1
	CBS 261.92	*M. piperita* (Lamiaceae)	GU237813.1	EU880905.1	GU237519.1	KY484736.1
*B. lilacis*	CBS 569.79		GU237892.1	EU880875.1	GU237498.1	KY484721.1
	CBS 489.94		KY484665.1	EU880876.1	MN983810.1	KY484720.1
*B. exigua* var. *rhapontica*	CBS 113651	*Rhaponticum repens* (Asteraceae)	KY484662.1	KY484566.1	KY484760.1	KY484713.1
*B. exigua* var. *rhapontica*	AvLM-Be	*Apocynum venetum* (Apocynaceae)	ON005131	OP611551	OP611552	OP611553
	PpBQ-Be	*Poacynum pictum* (Apocynaceae)	ON005132	OP611563	OP611564	OP611565
	PpBB-Be		ON005133	OP611554	OP611555	OP611556
	PpBH-Be		ON005134	OP611557	OP611558	OP611559
	PpBZ-Be		ON005135	OP611569	OP611570	OP611571
	PpBK-Be		ON005136	OP611560	OP611561	OP611562
	PpBX-Be		ON005138	OP611566	OP611567	OP611568
	Av-Be		ON005137	OP611548	OP611549	OP611550
*Phoma herbarum*	CBS 615.75		KF251212.1	EU880896.1	KF252703.1	KF253168.1

**Figure 4:**
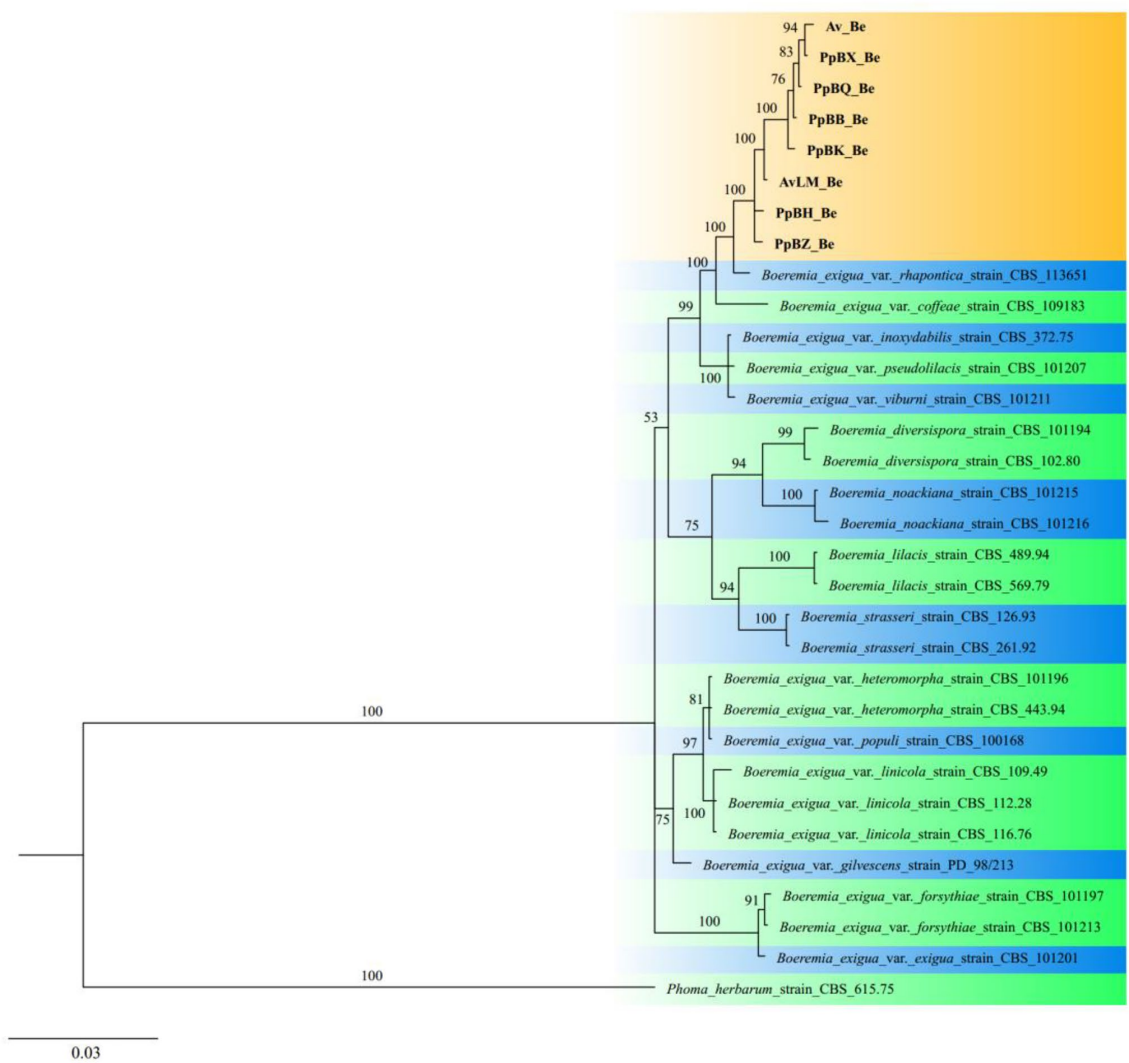
Phylogram generated for the *Boeremia* species from a Bayesian inference analysis based on the combined internal transcribed spacer region, the fragments of actin, β-tubulin and translation elongation factor-1 alpha genes. The tree was rooted to *Phoma herbarum* (strain: CBS 615.75).

In the single gene analysis, we found that the eight strains causing stem necrotic lesions disease on Luobuma could not be distinguished by the single loci *ITS* (Fig. S1), *ACT* (Fig. S2), and *TUB* (Fig. S3) but could be identified by loci *TEF* (Fig. S4). Moreover, the strains were closely related to *B. exigua* var. *rhapontica*, which was originally reported in *Rhaponticum repen*s. The phylogenetic tree (generated through multiple sequence alignment) also showed that the eight strains clustered with *B. exigua* var. *rhapontica* strain (Fig. 4). Based on these findings, the pathogen was identified to be *Boeremia exigua* var. *rhapontica*.

### Assessment of the disease development in the field

**Figure 5:**
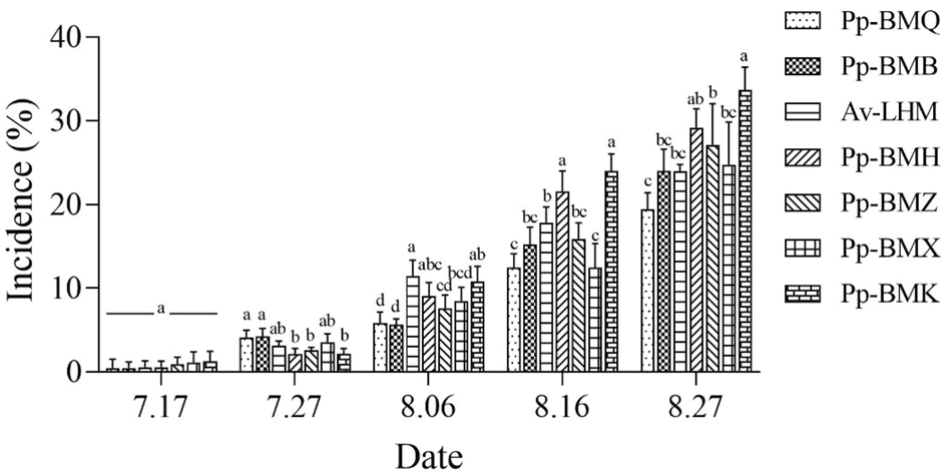
The incidence of seven ecotypes stem necrosis lesions disease caused by *B. exigua* var. *rhapontica* in 2020 in the field.

**Figure 6:**
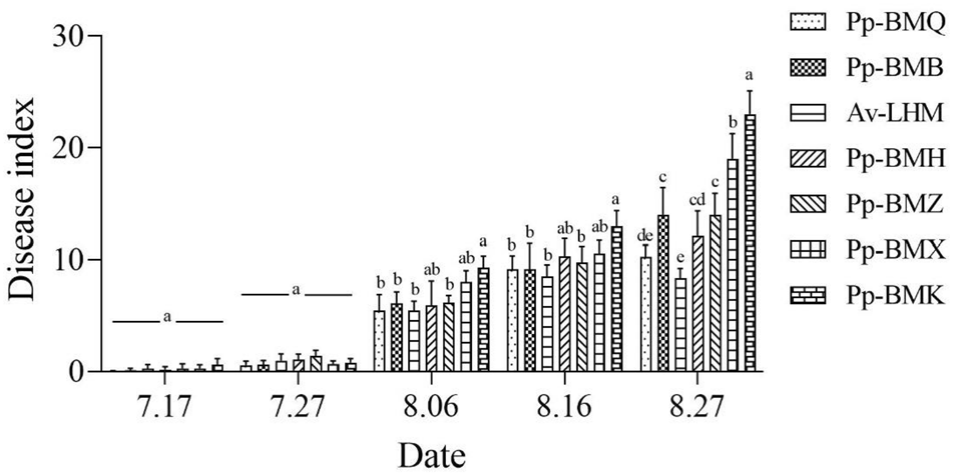
The disease index of seven ecotypes stem necrosis lesions disease caused by *B. exigua* var. *rhapontica* in 2020 in the field.

**Figure 7:**
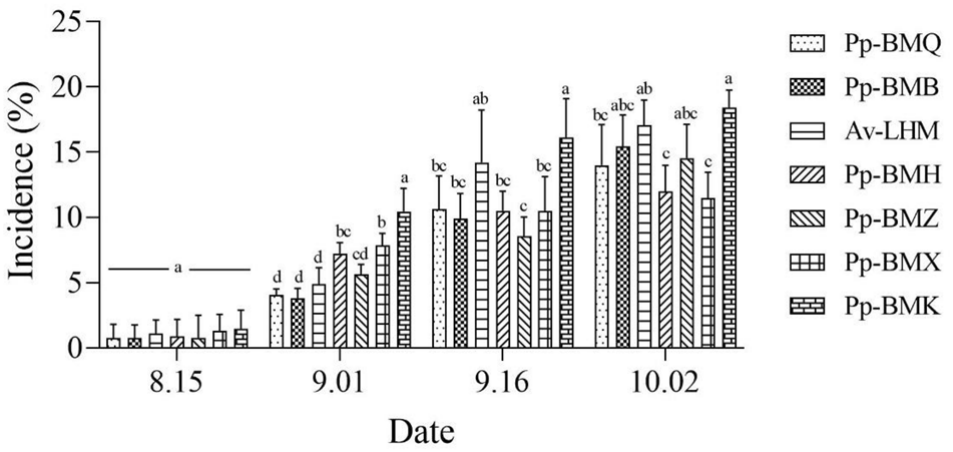
The incidence of seven ecotypes stem necrosis lesions disease caused by *B. exigua* var. *rhapontica* in 2021 in the field.

**Figure 8:**
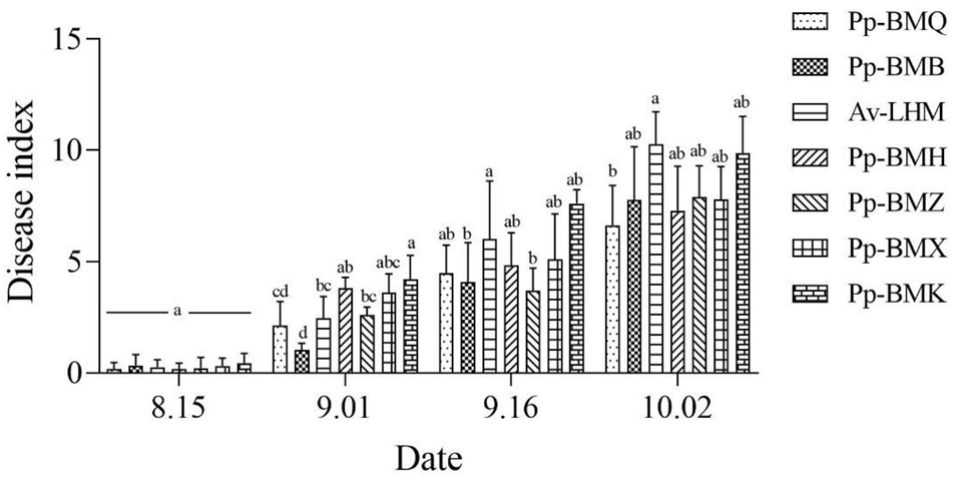
The disease index of seven ecotypes stem necrosis lesions disease caused by *B. exigua* var. *rhapontica* in 2021 in the field.

**Figure 9:**
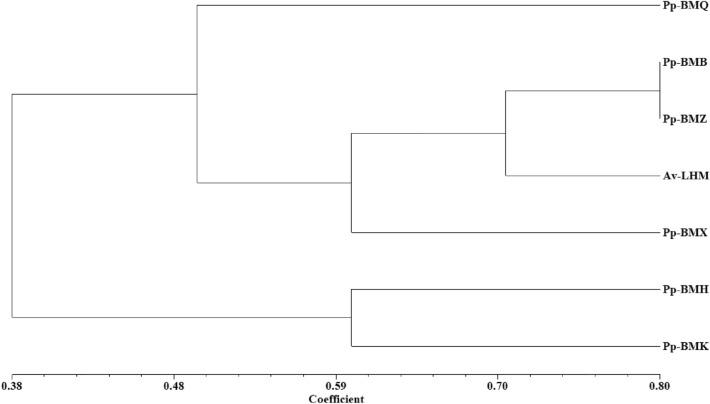
UPGMA (Unweighted pair-group method with arithmetic average) dendrogram generated from the incedence and disease index of seven ecotypes caused by *B. exigua* var. *rhapontica*. The cophenetic coefficient, r = 0.73.

The incidence and disease index of the seven ecotypes differed significantly (*P* < 0.05) in the field. The maximum incidence of the seven ecotypes ranged from 19.43% to 33.68%, and the maximum disease index ranged from 8.39 to 23.01 in 2020 (Figs. 5, 6), while in 2021, the ranges were 11.49% to 18.43% and 6.63 to 10.24 in 2021, respectively (Figs. 7, 8). Although the stem necrotic lesions of Luobuma first occurred from Mid-July to Mid-August in 2020–2021, the disease occurrence in 2020 was earlier and more severe than that in 2021. The disease index and incidence during the two-year growing season were the highest for Pp-BMK and Pp-BMH, and the lowest for Pp-BMQ (Fig. 9).

### Pathogenicity on the leaf tissues

**Figure 10:**
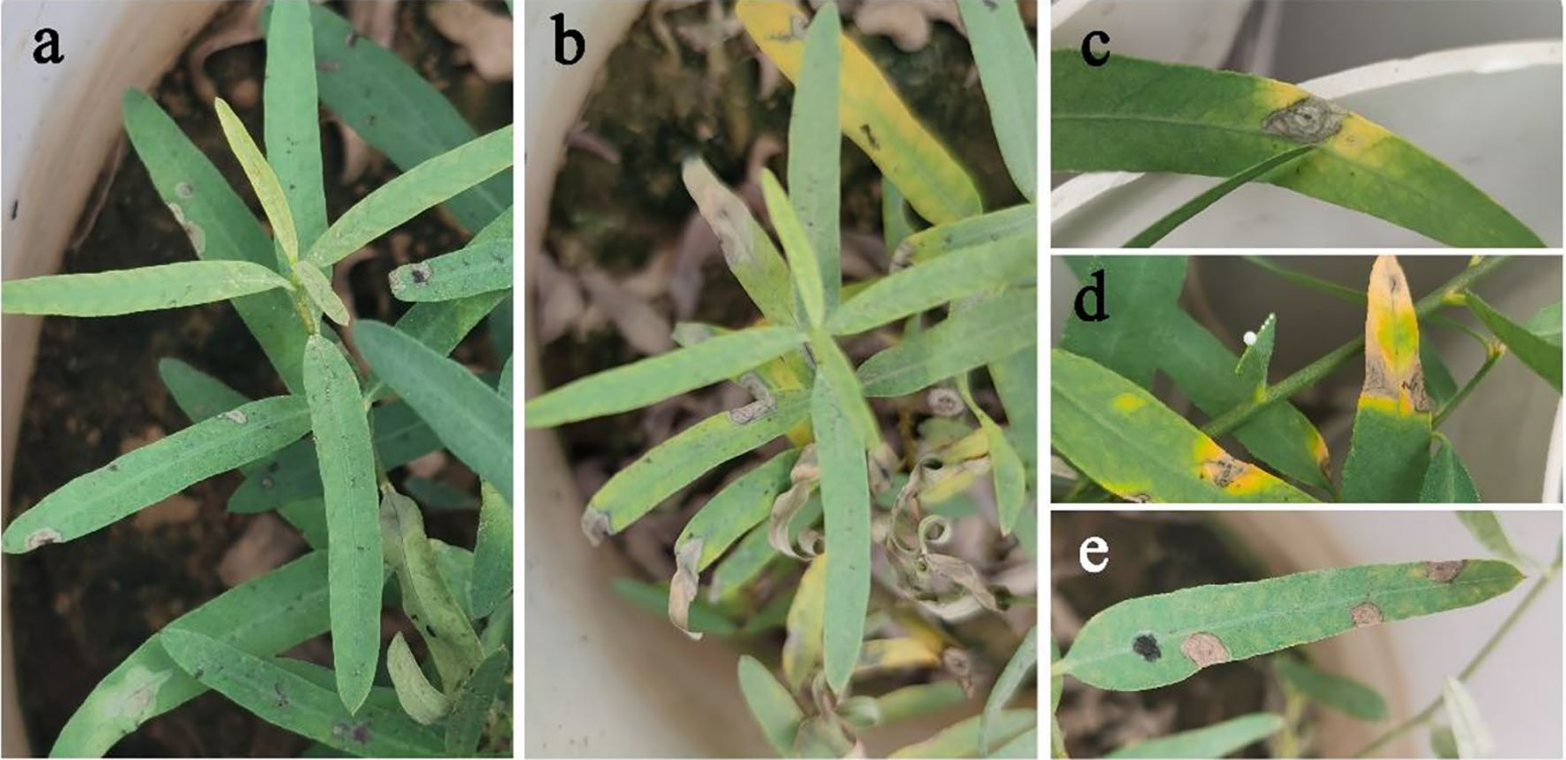
The symptoms of leaves necrotic lesions disease in inoculated leaves of Luobuma. (**a**) leaf symptoms of *A. venetum* 5 days after inoculation, (**b**) leaf symptoms of *A. venetum* 13 days after inoculation, (**c,d**) the clear wheel disease spots on the leaves of *P. hendersonni* and (**e**) unclear wheel disease spots on the leaves of *A. venetum* and *P. pictum*.

To explore whether *B. exigua* var. *rhapontica* infects leaves, we separately inoculated conidial suspensions of strains AvLM-Be and Av-Be on the leaves of the eight ecotypes. Five days after inoculation, the strains caused similar symptoms with *B. exigua* var. *rhapontica*, which resulted in discolored spots closer to the leaf edges and leaf tips (Fig. 10a). However, 13 days after inoculation, the necrosis lesions gradually expanded along the leaf margins and tips, and the disease spots presented unclear or clear concentric rings, which were irregularly shaped lesions after a few spots merged. This resulted in withering and death of some leaves (Fig. 10b). The leaves of *P. hendersonni* had clear concentric rings of the disease spots (Fig. 10c,d), while the leaves of *A. venetum* and *P. pictum* had unclear concentric rings (Fig. 10e). No symptoms were observed on the corresponding controls. Furthermore, the pathogen was re-isolated from the symptomatic leaves, and their identification was confirmed by morphology and molecular analysis. The pathogenicity analysis showed that under greenhouse conditions, *B. exigua* var. *rhapontica* could infect Luobuam leaves and induce slightly different symptoms for 8 ecotypes of the three species of Luobuma.

**Figure 11:**
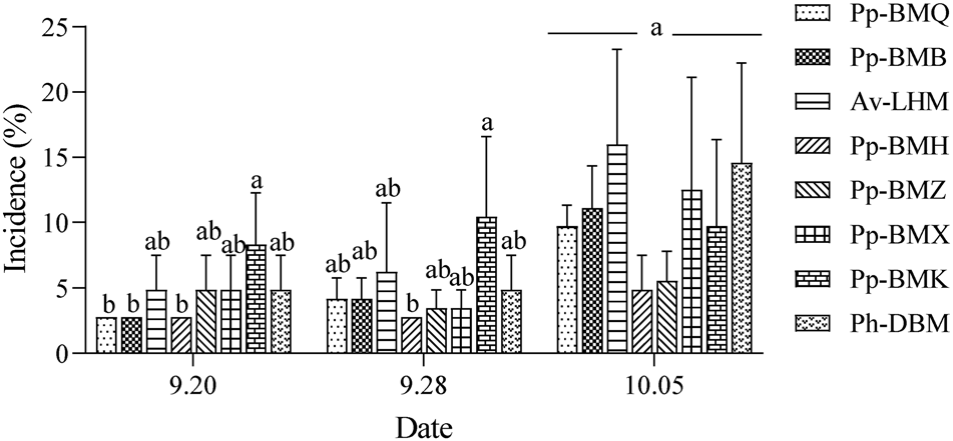
The disease incidence of eight ecotypes leaves inoculation with *B. exigua* var. *rhapontica* conidial suspensions under greenhouse conditions.

The leaf incidence of the eight ecotypes was investigated in triplicate from late September to early October under greenhouse conditions. The collected data showed that ecotype Pp-BMK was the highest incidence rate on Sept. 20 and Sept. 28 (8.34% and 10.42%, respectively), while the ecotype Pp-BMH had the lowest incidence rate (2.78% and 2.78%, respectively) (Fig. 11; *P* < 0.05). However, 21 days after inoculation, the incidence rate of ecotype Av-LHM was the largest (15.97%), while that of Pp-BMH was the lowest (4.86%).

### Pathogenicity on the stem tissues

**Figure 12:**
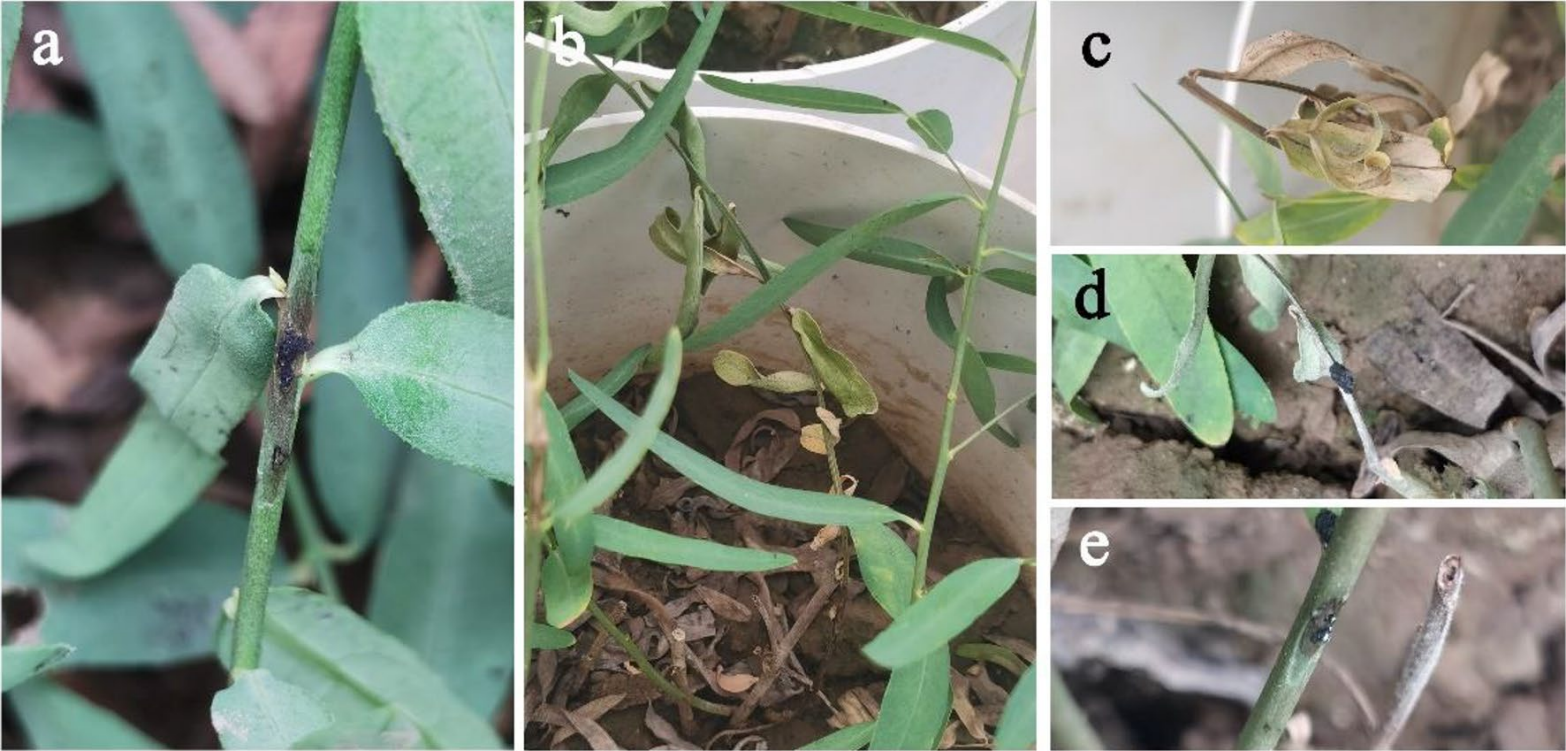
The symptoms of stem necrotic lesion spots disease in inoculated stems of Luobuma. (**a**) stem symptoms of *A. venetum* 5 days after inoculation, (**b**) stem symptoms of *A. venetum* 13 days after inoculation and (**c–e**) necrosis lesions on the stems of Luobuma.

To confirm Koch’s postulates, we inoculated the two strains (AvLM-Be and Av-Be) on the stem of the eight ecotypes. After 5 days, the stems of all inoculated ecotypes developed necrosis lesions, which had brown spots with wedge-shaped indentations at the beginning (Fig. 12a). However, 13 days after inoculation, the symptomatic stems and shoots withered and died (Fig. 12b–d). There were no differences in lesion shape on the stems of the eight ecotypes; however, the degree of necrosis lesion spots and the wedge-shaped indentations were more severe on the thick stems (Fig. 12a,e). No symptoms were observed on the corresponding controls. The morphological and molecular analysis of the re-isolated pathogen showed that the pathogen was *B. exigua* var. *rhapontica*. Moreover, pathogenicity tests showed that *B. exigua* var. *rhapontica* could infect stems of Luobuam under greenhouse conditions, and the resulting stem necrosis lesions shape was similar to those observed in the field; however, the lesion color differed from those under the field conditions.

**Figure 13:**
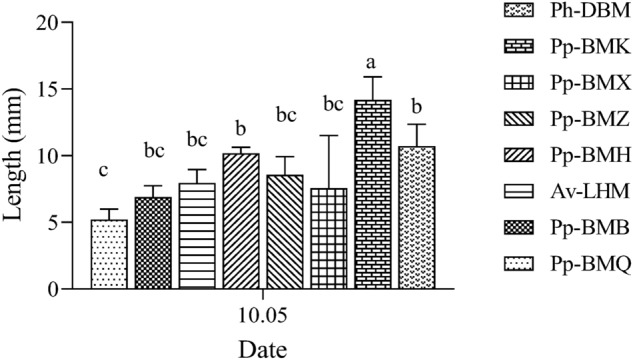
The length of necrosis lesions of eight ecotypes stems inoculation with *B. exigua* var. *rhapontica* mycelial plugs under greenhouse conditions.

The pathogenicity of *B. exigua* var. *rhapontica* had significant differences (*P* < 0.05) in stem tissues of the eight ecotypes. The necrosis lesion spot length of the eight ecotypes ranged from 5.20 mm (Pp-BMQ) to 14.18 mm (Pp-BMK) 13 days after inoculation. Therefore, these pathogenicity results showed that ecotype Pp-BMQ had higher resistance to the *B. exigua* strains, while ecotype Pp-BMK had lower resistance than the other ecotypes (Fig. 13).

## Discussion

The present study reported a novel and severe stem necrotic lesions disease on Luobuma caused by *Boremeria exigua*. The pathogenic fungus, *B. exigua*, also infects many other plants as their hosts. For example, 11 varieties of *B. exigua* reportedly infect 45 plant species belonging to 31 genera and 19 families^13^. Although the infection is host-dependent, the pathogen isolated from one host could also infect other different hosts. *B. exigua* var. *forsythiae* has previously been reported on *Forsythia* of the Oleaceae family in the Netherlands^6^ and has been shown to successively infect *Hydrangea macrophylla*^13^. Therefore, the nomenclature of the *Boeremia* varieties was based on different hosts and the combination of their morphology and molecules. In our study, *B. exigua* var. *rhaponticai* infected eight ecotypes belonging to three Luobuma species, which were differentiated based on the phylogeny of four loci (*ITS*, *ACT*, *TUB*, and *TEF*) coupled with their morphological characteristics. To our knowledge, this is the first report of *B. exigua* var. *rhapontica* causing stem necrotic lesions and leaf spots in Luobuma.

*Boeremia exigua* var. *rhapontica* has previously been identified at the species level on *Russian knapweed* of the family Asteraceae in Turkey in 2003^14^, and its variety *B. exigua* var. *rhapontica* was only identified in 2015 based on host association analysis^13^. In 2015, Berner^13^ described the colony, conidia, and pycnidia characteristics of *B. exigua* var. *rhapontica* isolated from Russian knapweed (*Rhaponticum repens*) on agar. *Boeremia exigua* var. *rhapontica* was found to be genetically closer to *B. exigua* var. *coffeae*, originally identified on coffee plants (*Coffea arabica*)^6,13^. However, the *B. exigua* var. *rhapontica* was proposed based on the molecular and biological characteristics analysis^13^. The present study cultured the *B. exigua* var. *rhapontica* on three different culture media (PCA, OA, and PDA), and its colony characteristics were described. The colonies had irregular whitish-grey lobed margins on PCA, OA, and PDA. The morphological characteristics of the conidia and hyphae on PCA were also described, and these descriptions provide an important reference for *B. exigua* var. *rhapontica* identification in future studies.

Previous studies recommended that *Boeremia* be identified based on a combined phylogeny analysis using *ITS*, actin, beta-tubulin, calmodulin, and translation elongation factor 1-alpha genes, rather than using any of the single genes^13,15^. Therefore, our study used the four genes (*ITS*, *ACT*, *TUB*, and *TEF*) to identify the strains. The phylogenetic tree showed that the eight strains clustered closely with *B. exigua* var. *rhapontica*. Moreover, in the single gene analysis, the strains were comparable with *B. exigua* var. *rhapontica* using only the loci *TEF*. Therefore, based on the molecular and biological data obtained in this study, the strains that infected the leaves and stems of three Luobuma species were identified as *B. exigua* var. *rhapontica*.

*Boeremia exigua* reportedly infects leaves^10,16,17^, fruits^17^, tubers^18^, roots^19^, and stems^10^ in the field. For instance, *B. exigua* has been reported as a leaf spot pathogen of Japanese ginseng (*Panax japonicus*) in China, affecting approximately 95% of plants in the field and causing severe leaves necrotic lesions with elliptical and flavicant margins, which are irregularly shaped at the leaf margins or tips^16^. The pathogen also causes leaves and fruit spots in okra (*Abelmoschus esculentus*) and results in brown to brownish black and sunken cavities lesions with an incidence rate of 35%–55% in different fields^17^. Other studies have shown that *B. exigua* causes underground plant diseases; for example, the pathogen reportedly causes tuber rots in sweet potatoes (*Ipomoea batatas*)^18^. *B. exigua* also causes black root rot of the industrial chicory (*Cichorium intybus* var. *sativum*) in Chile, exhibiting dark and firm sunken lesions, which turn into black-colored cavities on the crown, with yield losses of up to 31%^19^. The stem and leaf spot disease of the common speedwell (*Veronica officinalis*) is also caused by *B. exigua* in Switzerland^20^. The present study reported the first *B. exigua* var. *rhapontica* infection cases of *A. venetum*, *P. pictum*, and *P. hendersonni*. The pathogen infected Luobuma stems under natural conditions, but the leaves were not infected. However, the leaves inoculated with *B. exigua* var. *rhapontica* conidial suspensions showed visible symptoms of necrosis lesion spots with unclear or clear concentric rings, which caused withering and death of the Luobuma.

The absence of the symptomatic leaves in the field could be linked to Luobuma rust disease caused by *Melampsora apocyni*, an aggressive causal agent in the field, with an incidence rate of up to 90% and whose urediospores could cover the whole leaves in severe cases^21^. The rust disease of Luobuma usually occurs from late June to early July in Yuzhong; however, the stem necrotic lesions disease of Luobuma began in late July to early August. Therefore, *M. apocyni* or its metabolites could have occupied the infection sites; thus, *B. exigua* var. *rhapontica* had no space to invade. Meanwhile, the leaf disease caused by *B. exigua* var. *rhapontica* was not prevalent in the 2-year field study, and this necessitates further investigation. Furthermore, our results demonstrated that the different ecotypes had slight differences in their disease symptoms, especially in the color of the disease spots. This may be associated with the stem color of the different ecotypes^5^.

Since we observed that the pathogen *B. exigua* var. *rhapontica* was prevalent on Luobuma plants, including the two species (*A. venetum* and *P. pictum*), we further investigated the disease index and occurrence of the stem necrotic lesions disease on the seven ecotypes of the two species in the same fields in the year 2020 and 2021. This was to reveal the relationship between species or ecotypes and disease development. The results demonstrated that the effect of the pathogen on the two species had no difference at the species level but differed at the host plant (the ecotypes) level. Moreover, the pathogen was also isolated successfully from the soil, indicating that soil might be one of the overwintering pathogen sites, acting as a primary source of infection for the subsequent growing seasons.

The susceptibility of cultivars/ecotypes to *B. exigua* infection varied. For instance, potato (*Solanum sp.*) varieties had varying resistance levels to the invasion of *B. exigua* var. *foveata*^22^. A pathogenicity trial of the lima bean (*Phaseolus lunatus*) cultivars inoculated with *B. exigua* var. *exigua* also showed that the cultivars had different susceptibility levels to *B. exigua* var. *exigua*^23^. In our study, consistent differences in stem disease occurrence on the different ecotypes were observed in the greenhouse and field conditions. Stems and leaves of the different ecotypes exhibited different responses to *B. exigua* var. *rhapontica* infection.

## Conclusions

To the best of our knowledge, this is the first report of stem necrotic lesions and leaf spots disease on Luobuma caused by *B. exigua* var. *rhapontica*. The disease incidence rate in the field was up to 34%, and its prevalence and damage were second to the rust disease and spot blight caused by *Septoria apocyni*^24^. The present study provides useful information for identifying and determining the pathogenicity of *B. exigua* var. *rhapontica*, and the occurrence of the stem necrotic lesions disease. However, further studies are required to determine the control measure of the disease.

## Methods

### Site establishment

A seed-breeding field composed of 8 Luobuma ecotypes (Av-LHM, Pp-BMQ, Pp-BMB, Pp-BMH, Pp-BMZ, Pp-BMX, Pp-BMK, and Ph-DBM) was established in 2017 in Yuzhong County, China (35° 34′ 20″–36° 26′ 30″ N, 103° 49′ 15″–104° 34′ 40″ E). The seeds were provided by Altay Gaubao Tea Co., LTD., located in Altay Prefecture of the Xinjiang Uyghur Autonomous Region, China. Plant rows were 1 m apart, while the spacing within the rows was 0.5 m apart. Each ecotype had four plots (5 by 10 m^2^ each). Among the planted ecotypes, Ph-DBM degraded gradually and eventually died in 2019; thus, only seven ecotypes were studied.

### Disease survey

**Figure 14:**
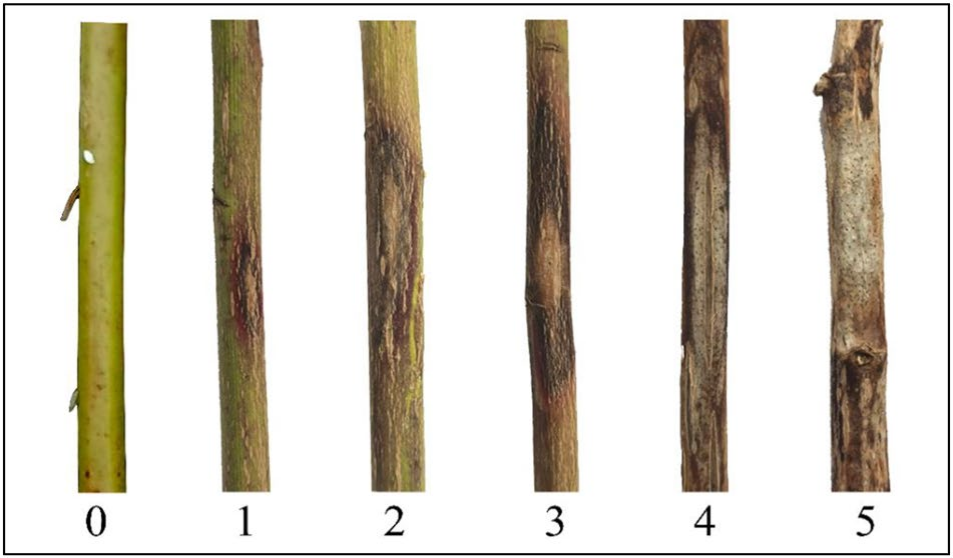
The severity levels of stem necrotic lesions were visually estimated from the stem symptoms.

We performed successive disease surveillance during the growth season of Luobuma from 2020 to 2021 to determine the field the incidence and disease index of stem necrotic lesions of the seven ecotypes. The disease symptom was observed and recorded, and the severity of the stem necrotic lesion was visually estimated as the percentage of observed stems covered by necrotic lesions with/without pycnidia. The disease severity scale included 0. for no infection signs; 1. for small reddish-brown disease spot; 2. for long strip or elliptical reddish-brown disease spot; 3. for necrotic lesions with larger black-brown-patched margins, the white or off-white centers with fewer pycnidia or without pycnidia; 4. for necrotic lesions with depression and larger white or off-white areas at the center with a mass of ripe pycnidia; 5. for necrotic lesions with girdling depressions and withered stems, or dead stems (Fig. 14). The incidence and disease index of the stem necrotic lesions were investigated in five locations for each ecotype. All stems from 5 Luobuma plants, one from each location, were analyzed for the symptoms, and those with many necrosis lesions per stem were recorded to have a maximum severity level.

### Sample collection and isolation

The stems were collected from the 7 ecotypes of Luobuma for the necrotic lesion symptoms analysis. The roots of the plants with severe stem necrotic lesions were also collected from ten plants of each ecotype to determine whether the pathogen also infected the roots. Two isolation methods were used in the present study to accurately identify the pathogen causing the stem necrotic lesions disease. Briefly, the infected stems and healthy root tissues were surface-sterilized in 75% ethanol for 45 s, rinsed thrice with sterile distilled water, and air-dried on sterile filter paper. Ten segments (0.4 cm × 0.3 cm) of the symptomatic stem and healthy root tissues were cut from 10 random plants and were cultured in a Petri dish containing potato dextrose agar (PDA) (one Petri dish per plant) and incubated at 23 ± 1 °C for 5 days. For the other isolation method, the pycnidia from stem tissues were cultured on PDA under the same conditions. To determine whether the pathogen exists in the soil, we collected soil samples (5 cm–10 cm soil layer) from four locations of each ecotype containing Luobuma with severe stem necrotic lesion symptoms. The soil samples were frozen at 4 °C for 2 weeks, and the soil microorganisms were isolated based on the plate dilution method^25^.

### Morphological and biological characteristics analysis

The mycelial plugs (4 mm diameter) from the edge of the growing colonies were excised and cultured in a fresh media (PCA, OA, and PDA) and incubated at 25 °C in the dark for 3 weeks. The morphological characteristics, including the colony colour and outline, mycelium structure, spores size, and the presence/absence of pycnidium, were determined using 3-week-old cultures. The spore size was measured for three strains using an Olympus BX-51 microscope (Olympus Corporation, Japan) at × 50 magnification. Moreover, the same microscope was used to observe the spore and mycelium structures. Relevant data were compared via Tukey’s test (*P* < 0.05) using IBM SPSS Statistics 19.0.

We also measured the effect of temperature on the pathogen growth after culturing the plates containing the mycelial plugs (4 mm diameter) were incubated at 5 °C, 10 °C, 15 °C, 20 °C, 25 °C, 30 °C, and 35 °C in the dark for 3 weeks. Colony diameter (4 replicates) was measured after 10 days using the method by Han et al.^26^.

### DNA extraction, polymerase chain reaction (PCR) amplification, and sequencing

Total genomic DNA was extracted from mycelia (about 60 mm diameter), scraped from one-week colonies cultured on PDA, using Fungal Mini Kit (Omega bio-Tek, Doraville, CA), according to the manufacturer’s protocol with slight modifications. The purity and concentration of the obtained DNA were assessed using a NanoDrop ®ND-1000 UV (NanoDrop Technologies, USA), and the DNA was stored at − 20 °C for further analysis. The presence of rDNA internal transcribed spacer (*ITS*) region, actin (*ACT*), β-tubulin (*TUB*), and translation elongation factor-1 alpha (*TEF*) genes in the extracted DNA was determined by conventional PCR. The sequences of primers used are displayed in Table 3.

**Table 3:** The primers used in the study.

Primer Name	Sequences	Literatures
ITS1/ITS4	5′-TCCGTAGGTGAACCTTGCGG-3′ 5′-TCCTCCGCTTATTGATATGC-3′	White et al.^27^
ACT-512F/ACT-783R	5′-ATGTGCAAGGCCGGTTTCGC-3′ 5′-TACGAGTCCTTCTGGCCCAT-3′	Carbone and Kohn^28^
BT2A/BT2B	5′-GGTAACCAAATCGGTGCTGCTTTC-3′ 5′-AACCTCAGTGTAGTGACCCTTGGC-3′	Glass and Donaldson^29^
EF1-728F/EF1-986R	5′-CATCGAGAAGTTCGAGAAGG-3′ 5′-TACTTGAAGGAACCCTTACC-3′	Carbone and Kohn^28^

The PCR reactions were conducted on a DLAB PCR TC1000-G Thermo cycler in a final volume of 25 μL, which included 9 μL of ddH_2_O, 1 μL of each primer (forward and reverse), 1.5 μL of the template DNA, and 12.5 μL of DNA polymerase. The PCR amplification process consisted of an initial denaturation at 95 °C for 5 min followed by 35 cycles of denaturation at 95 °C for 30 s, a 2 min annealing at 53 °C for *ITS*, 56 °C for the *ACT*, 57 °C for *TUB*, and 55 °C for *TEF*, elongation at 72 °C for 2.5 min, and extension at 72 °C for 30 s. The final extension was at 72 °C for 8 min. The effectiveness of the PCR and the length of the amplified DNA fragments were verified by 1.5% agarose gel electrophoresis. DNA sequencing was then performed by Sangon Biotech (Wuhan, China).

### Phylogenetic analysis

Sequences of all four target regions (*ITS*, *ACT*, *TUB*, and *TEF*) obtained from *Boeremia* species were individually aligned using the Clustal X program of the MEGA software version 7, and the parameters were manually adjusted to allow for maximum sequence similarity. Then the four loci were combined using the Sequence Matrix 1.8. Bayesian inference trees were then constructed using MrBayes v3.2. The Markov chain was run for a maximum of 1 million generations, in which every 50 generations were sampled and the first 25% of Markov chain Monte Carlo (mcmc) samples were discarded as burn-in.

### Pathogenicity tests

The pathogenicity test was conducted using the pot experiment. The seeds of three Luobuma species used in the present study were collected in 2016 from a nursery comprising eight ecotypes, established in 2009 at Alakak township, Xinjiang Uyghur Autonomous Region, China (87° 33′ 50′′ E, 47° 42′ 41′′ N). The 8 ecotypes are the same as those grown in Yuzhong, Gansu Province. The seeds were sterilized with 75% alcohol and germinated in Petri dishes containing two layers of sterilized filter papers, previously soaked in 5 mL of sterile distilled water. Five days after germination, the seedlings were transplanted to planting trays containing vermiculite, which was autoclaved twice at 121 ℃ for 6 h. In April 2019, the seedlings were transplanted into pots (20 cm diameter and 60 cm height and made of artificial PVC pipe material) containing a mixture of loess, sand, and rotten chicken manure (volume ratio, 10:3:1). Each pot was transplanted with 4 seedlings, and the average greenhouse temperature was 25 °C during the day and 18 °C at night, while the average relative humidity (RH) was 72% during the day and 81% at night.

Since the six strains isolated from infected Luobuma stems had the same morphological and biological characteristics, we used AvLM-Be isolated from symptomatic Luobuma stems as a representative strain for Koch’s postulates analysis. Additionally, the Av-Be strain isolated from the soil was also used in the present study to determine the pathogenicity of the soil strains. The two strains (AvLM-Be and Av-Be) were cultured on PCA at 25 °C for one month to produce the pycnidium. Two groups of the pathogen inoculums were then re-prepared for inoculating the leaves and stems of Luobuma plants grown 60 days in pots. The inoculum suspension containing 1 × 10^7^ spore was mixed with two drops of Tween 80, and 20 mL of the spore suspension was sprayed on the leaves of the eight Luobuma ecotypes. Four pots were set for each ecotype. The other pathogen inoculum was filtered by two layers of sterilized gauze, and the hypha segments and the colonized medium particles trapped on the gauze were used to inoculate the Luobuma stems. Three holes, about 0.2 mm depth, were created on the lower parts of the stems using a 0.3 mm diameter needle and were inoculated by placing the hypha segments or the colonized medium particles on the wounded parts of the stems. The plants were then covered with black plastic bags for 48 h after inoculation. After 5 days, the lengths of superficially visible stem necrotic lesions were measured, and the disease incidence of leaves was assessed and recorded.

### Data analysis 

The incidence and disease index data were analyzed using a numerical taxonomy and multi-variate analysis systems NTSYS-pc 2.1b (Exeter Software, Setauket, New York, USA), to clarify the resistant and susceptible ecotypes, as described by Villarino et al.^30^.

The significant ranges were: confirmed incidence of the stems necrosis lesions on Luobuma (< 1%; 1–2%; 2–3%; 3–4%; 4–5%; 5–6%; 6–7%; 7–8%; 8–9%; 9–10%; 10–11%; 11–12%; 12–13%; 13–14%; 14–15%; 15–16%; 16–17%; 17–18%; 18–19%; 20–21%; 21–22%; 22–23%; 23–24%; 24–25%; 25–26%; 26–27%; 27–28%; 28–29%; 29–30%; 30–31%; 31–32%; 32–33%; 33–34%; 34–35%); disease index of the stems necrosis lesions on Luobuma (< 1; 1–2; 2–3; 3–4; 4–5; 5–6; 6–7; 7–8; 8–9; 9–10; 10–11; 11–12; 12–13; 13–14; 14–15; 15–16; 16–17; 17–18; 18–19; 20–21; 21–22; 22–23; 23–24; 24–25).

## Supplementary Information

Supplementary Figures.

## Data Availability

All data generated or analyzed during this study are included or specifed in this published article. The seeds were provided by Altay Gaubao Tea Co., LTD., located in Altay Prefecture of the Xinjiang Uyghur Autonomous Region, China. Plant materials were collected according to the relevant institutional, national, and international guidelines and legislation.
