# Supplementary material for: Characterization of Boeremia exigua causing stem necrotic lesions on Luobuma in northwest China
Source: Sci Rep. 2022 Dec 14;12:21609. doi: 10.1038/s41598-022-25125-1 (PMC9751102; doi:10.1038/s41598-022-25125-1)
Supplement: Supplementary file 1 — Supplementary Figures. [file 41598_2022_25125_MOESM1_ESM.docx]

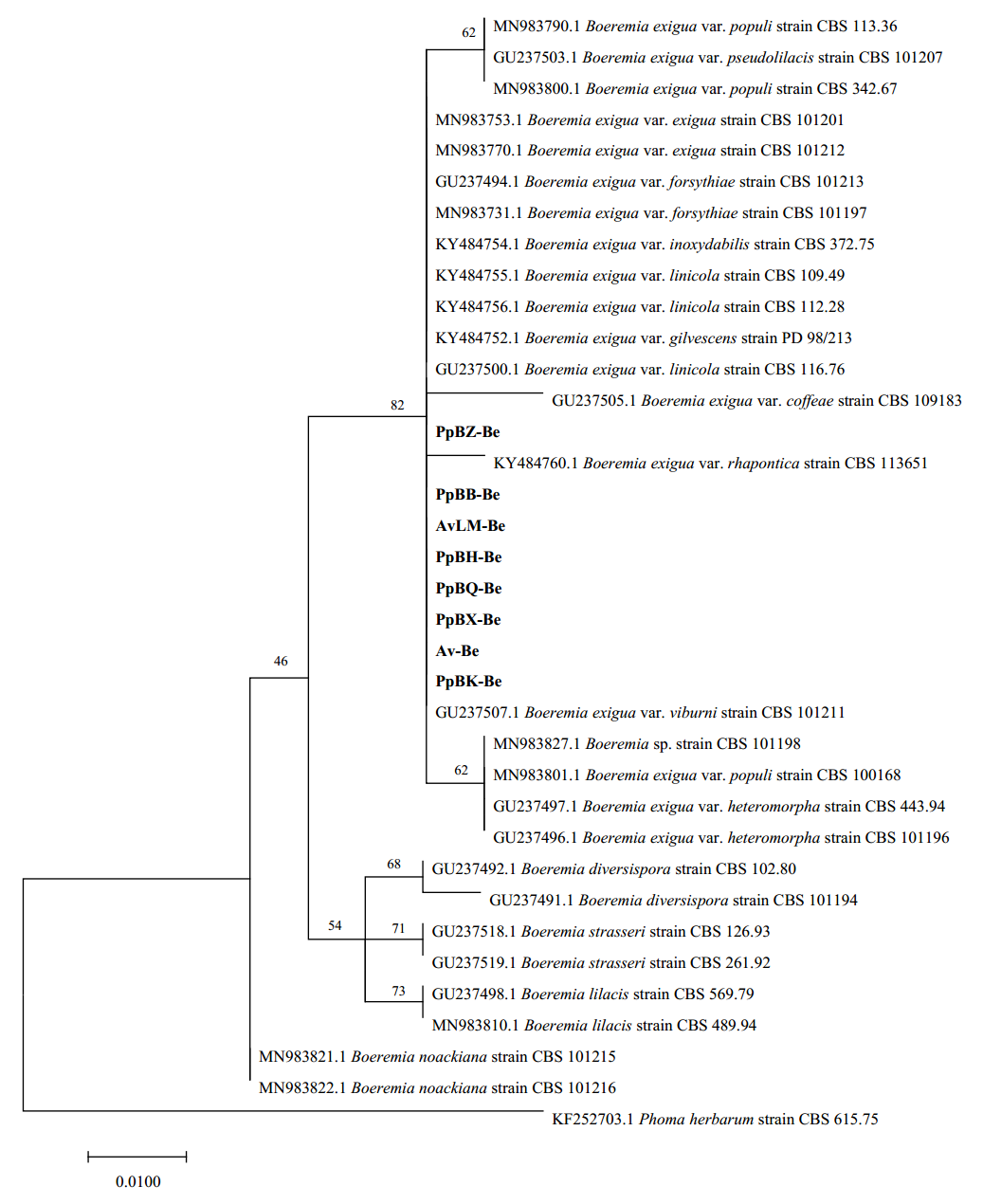


**Fig. S1** Phylogram generated for the *Boeremia* species from a maximum likelihood (ML) analysis based on β-tubulin. The tree was rooted to *Phoma herbarum* (strain: CBS 615.75)


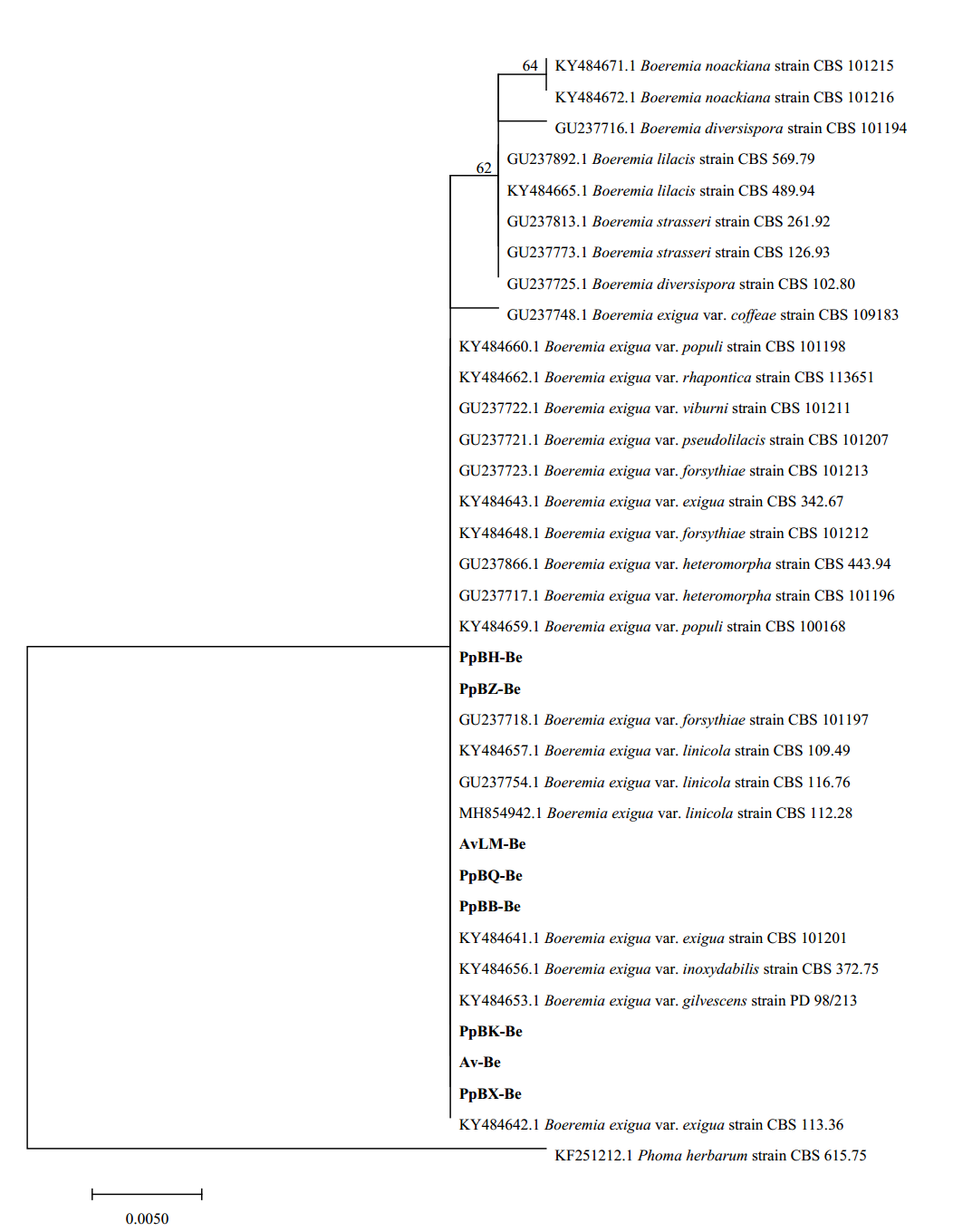


**Fig. S2** Phylogram generated for the *Boeremia* species from a maximum likelihood (ML) analysis based on the internal transcribed spacer region. The tree was rooted to *Phoma herbarum* (strain: CBS 615.75)


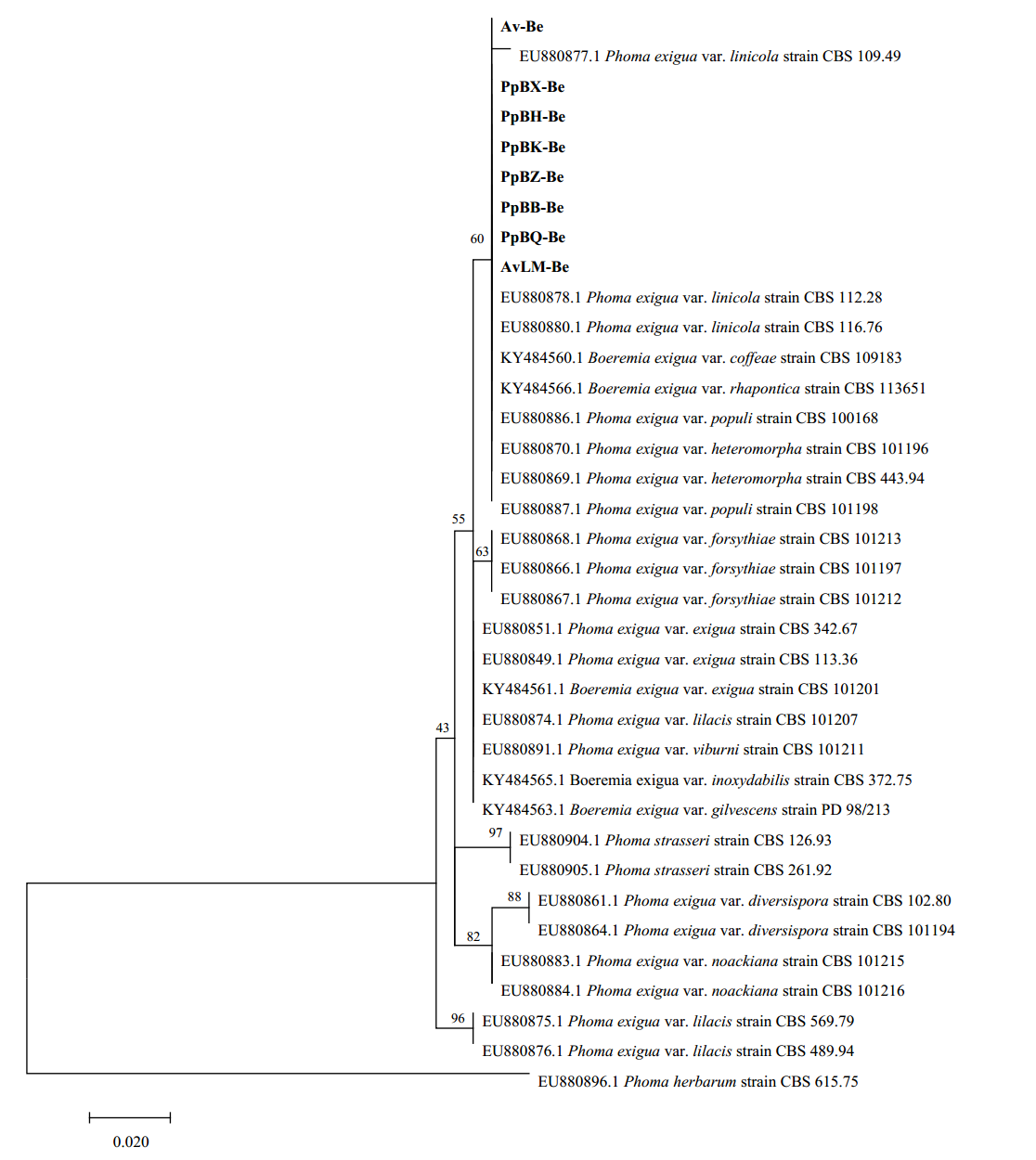


**Fig. S3** Phylogram generated for the *Boeremia* species from a maximum likelihood (ML) analysis based on the fragments of actin. The tree was rooted to *Phoma herbarum* (strain: CBS 615.75)


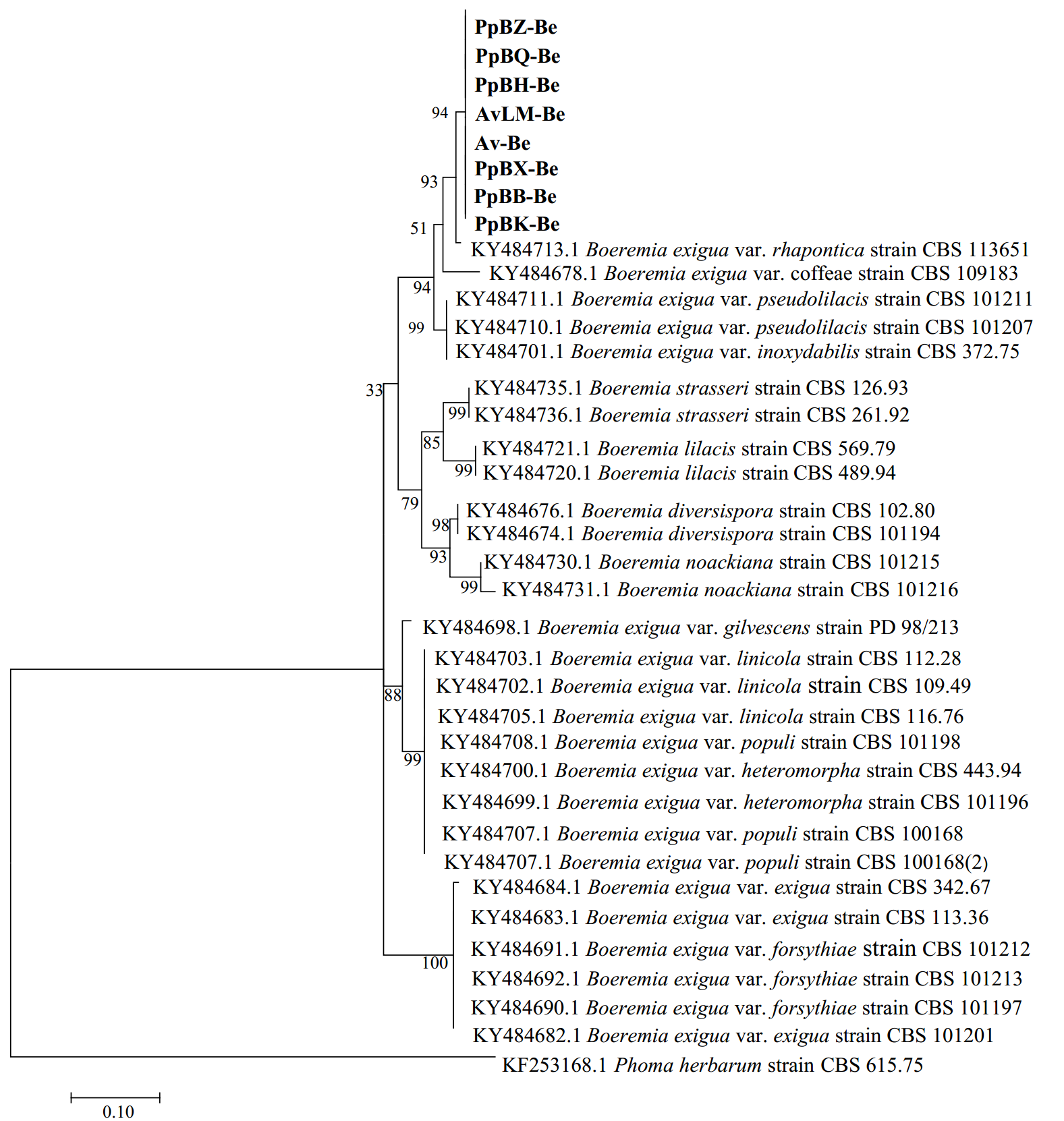


**Fig. S4** Phylogram generated for the *Boeremia* species from a maximum likelihood (ML) analysis based on translation elongation factor-1 alpha genes. The tree was rooted to *Phoma herbarum* (strain: CBS 615.75)
